# Supplementary material for: Analysis of PPARγ Signaling Activity in Psoriasis
Source: Int J Mol Sci. 2021 Aug 10;22(16):8603. doi: 10.3390/ijms22168603 (PMC8395241; doi:10.3390/ijms22168603)
Supplement: Supplementary file 1 [file ijms-22-08603-s001.zip › Supplemental materials_Analysis of PPARg signaling activity in psoriasis/Pathway models/Models images and html files/PPARG inhibits phenotypes assotiated with psoriasis/241954.html]

depressive symptoms


# Disease depressive symptoms

|  |  |
| --- | --- |
| URN | urn:agi-meshdis:Depressive%20Symptoms |
| Total Entities | 0 |
| Connectivity | 2229 |
| Name | depressive symptoms |

---

|  |  |
| --- | --- |
| Pathway | PPARG inhibits phenotypes assotiated with psoriasis |

---

|  |  |
| --- | --- |
| MedScan ID | 9006029 |

---

|  |  |
| --- | --- |
| Alias | Symptoms of Depression |
|  | Depressive Symptoms |
|  | Symptoms, Depressive |
|  | Depressive mood |
|  | Depression, Emotional |
|  | Depressive Symptom |
|  | Symptom, Depressive |
|  | Depressive moods |
|  | Depression (Emotion) |
|  | depressed mood |
|  | depressed moods |
|  | Depressions, Emotional |
|  | Emotional Depression |

---

|  |  |
| --- | --- |
| MeSH ID | M0006029 |

---

|  |  |
| --- | --- |
| MeSH Heading | Depressive Symptoms |

---
